# Secondary cervical elongatio due to large Gartner cyst: A rare case

**DOI:** 10.1016/j.ijscr.2020.05.054

**Published:** 2020-05-30

**Authors:** Surahman Hakim, Yulia Margaretta Sari, Achmad Kemal Harzif

**Affiliations:** a Urogynecology Division Department of Obstetrics and Gynecology, Faculty of Medicine University of Indonesia/Dr. Cipto Mangunkusumo Hospital, Jakarta, Indonesia; b Endocrinology and Fertility Division Department of Obstetrics and Gynecology, Faculty of Medicine University of Indonesia/Dr. Cipto Mangunkusumo Hospital, Jakarta, Indonesia

**Keywords:** Large Gartner cyst, Elongatio coli, Manchester forthegill

## Abstract

**Introduction:**

The exact mechanism by which pelvic tissues lose their anatomic support and undergo descent through the genital hiatus remains elusive. In this case, large Gartner cyst causes secondary cervix elongatio due to the weight of the cyst. Gartner cyst arise from the remnants of the mesonephric ducts. They are typically located in the anterolateral wall and are small. The rarity of this case is very interesting because of the mass size reaches 15 cm and causes cervical elongation.

**Presentation of case:**

This article report a case of 37 years old women who complained large mass came out from vagina. Patient was diagnosed with large anterior vaginal cyst and cervical elongatio. Ultrasound finding suggested that the cyst was originated from anterior wall of vagina in accordance with Gartner cyst. Patient underwent cyst excision, there was no connection between the cyst and urethra. Operation was continued with Manchester Forthegill to restore normal anotomy of the cervix.

**Discussion:**

Secondary cervical elongatio in this case was caused by large Gartner cyst, eventhough there are possibility involvement of other factors. Cervical elongatio plays important role in the decision of operation in this case after removal vaginal cyst.

**Conclusion:**

Secondary cervix elongation due to Large Gartner cyst is rare condition. Enlarge cyst causes secondary cervix elongatio, and operation technique was also challenging.

## Highlights

• Secondary cervix elongation due to Large Gartner cyst is rare condition.
• Enlarge cyst cause secondary cervix elongation.
• Manchester operation technique in this case was also challenging.

## 1 Introduction

Pelvic organ prolapse is a growing gynecologic problem because of the increased life expectancy of women. Pelvic organ prolapse may includes downward descent of the vaginal wall and/or uterus. In general, 40% women with POP have cervical elongation. The extent of cervical elongation is proportionate with the degree of uterine descent. Cervix may elongate either in the supravaginal or infravaginal part. Supravaginal elongation is commonly associated with uterine prolapse, whereas infravaginal elongation is congenital. It is uncommon for the cervix to elongate until 10 cm in length. Cervical elongation plays an important role in the decision between hysterectomy and uterine preservation during genital prolapse surgeries. In recent years, uterine sparing procedures are becoming more common, though hysterectomy has classically played a role in pelvic floor. General risk factors for the development of POP include parity, age, obesity, genetic factors, and surgery, but specific pathways that may be involved in prolapse remain elusive [1,2].

The prevalence of vaginal cysts is estimated to be 1 in 200 women; however, this number is probably inaccurate, since most cysts are not reported. They are more common in women in their third and fourth decades and are usually detected incidentally. When symptomatic, patients may present with mild vaginal discomfort, vaginal pressure or fullness, vaginal mass or swelling, dyspareunia, vaginal bleeding, or urinary symptoms [2].

Gartner cyst arise from the remnants of the mesonephric ducts. They are typically located in the anterolateral wall and are small (ranging in size from 0.1 to 4 cm), but rarely > 10 cm. The cysts are lined by cuboidal or low columnar nonmucinous epithelium. In most cases, Gartner cyst is asymptomatic, less than 2 cm in size and is accidentally found during routine gynaecological examination. The cyst may enlarge and become symptomatic and/or compress surrounding organs like the bladder, urethra or colon [[3], [4], [5]].

This case is very interesting because of the Gartner cyst size reaches 15 cm and causes cervical elongation.

This case report has been reported in line with the SCARE criteria [6].

## 2 Patient information

A 37 years old woman complained protrussion mass which came out from vagina since 14 years ago. Initially the mass was small and the mass become enlarge when she was pregnant. As her pregnancy close to her due date the mass become larger. After delivery patient couldn’t insert the mass into vagina. Patient felt the mass become largest since 3 month ago and patient got difficulties to do activities. Micturition and defecation were normal.

## 3 Clinical finding

**Fig. 1:**
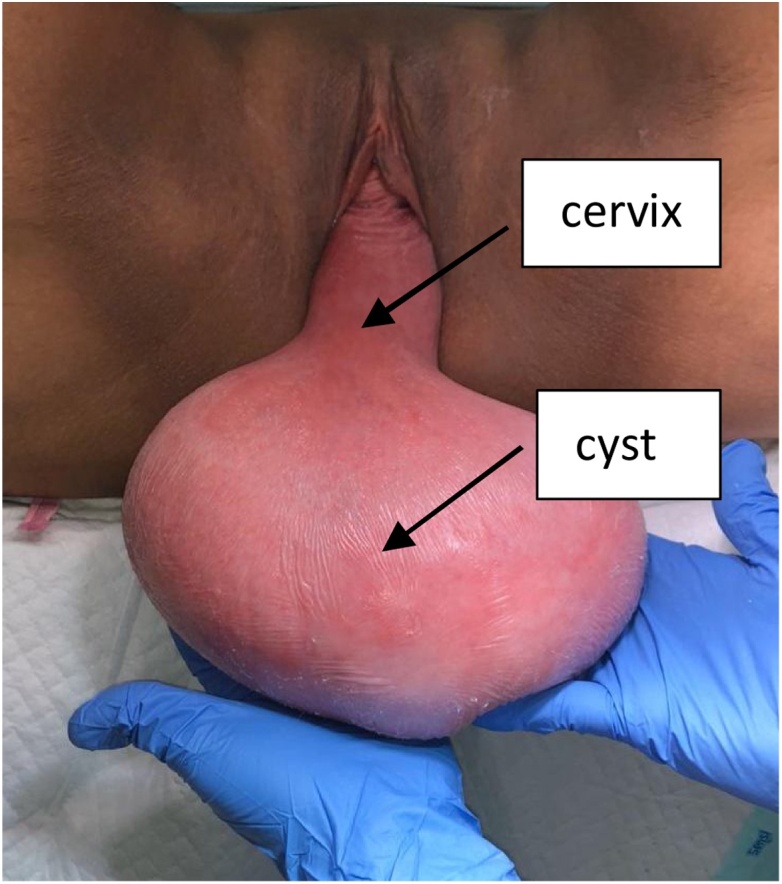
Preoperative Examination.

Physical examination showed 18 × 11 × 5 cm mass outside hymenal ring, with smooth vaginal mucosa and portio, with uterine sondage 15 cm. Cystic mass size 18 × 11 × 5 cm was palpated in anterior vaginal mucosa, smooth surface, fluctuated, without pressure pain. Uterus and adnexas was normal. Pelvic Organ Prolaps Quantification examination revealed that point Aa and Ba were +2, point C was +6, point Ap and Bp were −3, point D was 0. Genital hiatus was 7, perineal body was 4, and total vaginal length was 4. Rectal examination was within normal limit (Fig. 1).

## 4 Diagnostic assesment

**Fig. 2:**
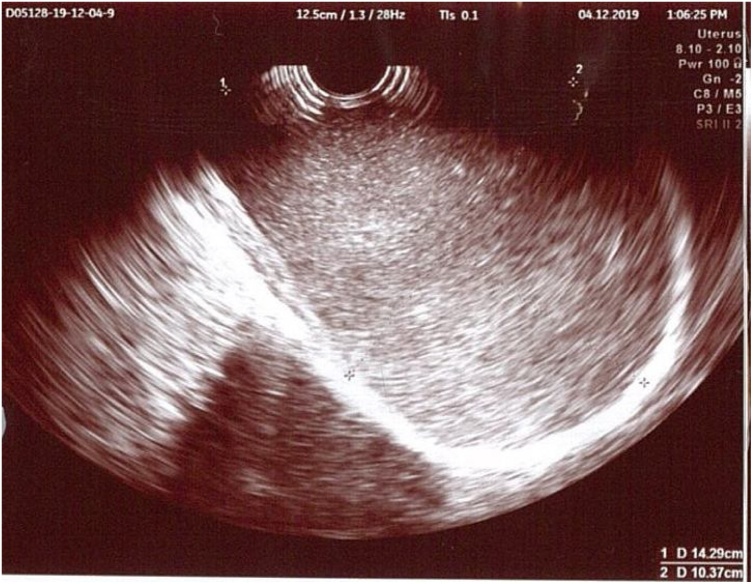
Ultrasonography Examination: Cystic mass with firm border contained coarse echointerna material.

Ultrasonography showed cystic mass with firm border contained coarse echointerna material. Mass with size 150 × 97 mm didn’t contain solid and vascular part. Mass was separated from canalis cervicalis originated from anterior vagina (area around anterior fornics and came out to vagina). Uterus size and shape were normal, and more caudal position (retracted by mass). Miometrium was homogen. Uterine cavity didn’t contain abnormal mass. Regular stratum basale endometrium, with thickness 4 mm. Cervix lengthwise to caudal (possible because retracted by mass). Cervical canal was normal with orificium uteri externum in posterior cystic mass. Both ovaries shape and size were normal. No abnormality was found in both adnexas. Conclusion: Cystic neoplasm of anterior vagina with secondary uterine prolapse (uterus and cervix were retracted caudally). No sign of malignancy (Fig. 2).

## 5 Therapeutic intervention

**Fig. 3:**
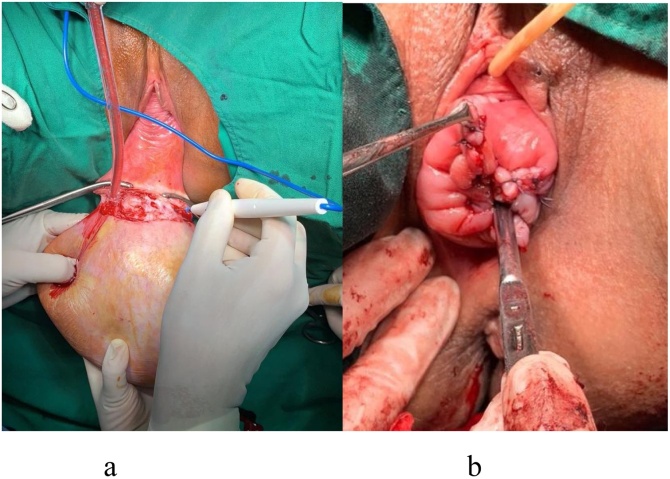
a) Excision was performed in proximal part (b) Manchester Forthegill procedure.

Excision of Gartner cyst and Manchester Forthegill operation was performed to restore normal anatomy. Uterus preservation considered to be appropriate choice because patient still want to have future fertility (Fig. 3).

## 6 Follow up and outcome

**Fig. 4:**
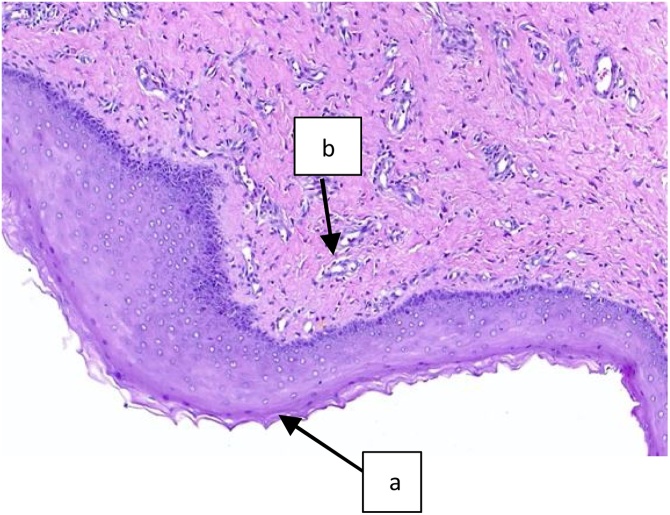
Microscopic view: Arrow (a) cyst wall was formed by stratified squamous epithelium keratinized; Arrow (b) connective tissue with chronic inflammation lymphocyte an plasma cell.

**Fig. 5:**
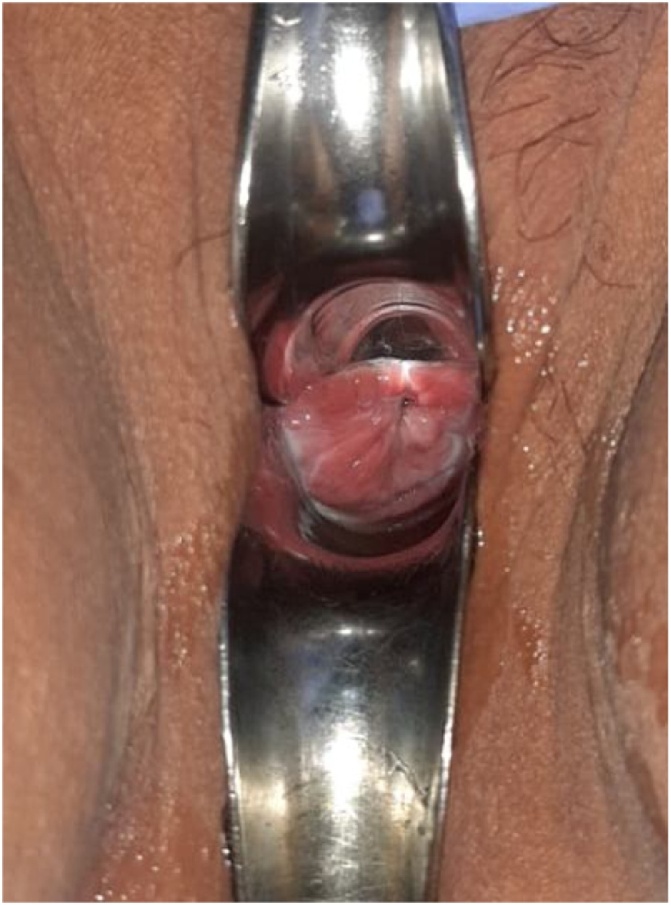
a) Physical examination a month after procedure.

Histopathology result found cyst wall was formed by stratified squamous epithelium keratinized and connective tissue with chronic inflammation lymphocyte an plasma cell not contradicting with Gartner cyst and cervical elongatio and malignancy was not found (Fig. 4). Post operative period was uneventful, and patient was discharged in satisfactory condition (Fig. 5).

## 7 Discussion

The exact mechanism by which pelvic tissues lose their anatomic support and undergo descent through the genital hiatus remains elusive. The “connective tissue deficiency” hypotheses are plausible since the cervix, vagina, uterosacral ligaments, and periurethral tissues are known to contain collagen, elastin, and smooth muscle. Collagen and elastin are important constituents of the extracellular matrix of the cervix. Collagen predominates, with type 1 collagen being the most abundant form (70%) and type 3 making up 30% of total collagen [2].

Biomechanical studies of the stretch and tension relationships of vaginal tissue have shown increased stretch ability of the vaginal skin with prolapse. It is not clear if these findings were confounded by age-related tissue changes. Worsening prolapse was associated with increased extensibility of the vaginal skin, but this finding was felt to be a result of prolapse rather than a direct cause [2].

Distal mesonephric duct in the female may persist as vestigial remnants in the anterolateral vaginal wall down to hymen as Gartner duct. The cyst developed as a result of secretory activity. Gartner ducts are identified in approximately 25% adult women and 1% evolve into Gartner duct cyst. Classically Gartner cyst was solitary, unilateral, and less than 2 cm in diameter. Large Gartner cyst can cause dyspareunia and other symptoms [7].

In this case, secondary cervical elongatio was caused by large Gartner cyst. The elongation affect vaginal part of the cervix. The large size of Gartner cyst causes cervical elongation in this patient eventhough there are possibility involvement of other factors [8].

Whether or not to preserve the prolapsed uterus is still a matter of debate. Furthermore, in recent years, more emphasis on the uterosacral ligaments as the most prominent structures to prevent uterine or middle compartment descensus has emerged. Therefore, it appeared logical to use techniques that incorporate these ligaments in the restoration of the middle compartment. Cervical amputation is part of the classical Manchester procedure, a surgical procedure for the correction of a prolapsed uterus [9].

Then cyst was excised in the proximal part. After cyst removal, reevaluation was performed to confirm cervical elongatio. Uterine sondage was 13 cm, operation was continued with Manchester Forthegill procedure. Longitudinal incision was perfomed in anterior and posterior vaginal wall. Vaginal wall was separated sharply and bluntly. And then amputation of cervix was performed. Forthegill technique was performed to tied bilateral cardinale ligament into anterior cervix. Stumdorf technique was performed with hegar dilator number 6 as guidance intra cervical canal. With needle pick up anterior lip or the stump remaining after amputation, 0,5 cm to the right or the midline, and bring the needle out through cervical canal. Afterward suture was is carried out through vaginal mucosa. The suture is carefully tightened with each stitch, thus causing the wound to become gradually covered by vaginal mucosa. The suture is tightened as strongly as possible and both ends are tied together tightly. Uterine sondage after operation was 8 cm. Manchester forthegill technique was chosen to preserve uterus and reproductive function.

Cervical elongatio plays important role in the decision of operation in this case after removal vaginal cyst. However, cervix may elongate postoperatively after uterine preservation. Preoperative assessment of cervical elongatio in this patient should be confirmed intraoperative after removal of the cyst. Because preoperative assessment can be inaccurate since the large cyst retract the cervix downward. Rare large Gartner cyst and cervical elongatio has aroused the interest to report this case, as Gartner duct cyst commonly present in small size.

## 8 Conclusion

Secondary cervix elongation due to Large Gartner cyst is rare condition. Enlarge cyst causes secondary cervix elongatio, and operation technique was also challenging.

## Funding

Source of funding is form the author only. There is no involvement of sponsor.

## Ethical approval

Because this is the case report and it is not a research, ethical approval was not required.

## Consent

Written informed consent was obtained from the patient for publication of this case report and accompanying images. A copy of the written consent is available for review by the Editor-in-Chief of this journal on request.

## Author contribution

Surahman Hakim: concept, operator, data analysis, final approval

Ahmad Kemal Harziq: drafting and revising

Yulia Margaretta Sari: data collection, data analysis, writing the paper

## Registration of research studies

NA.

## Guarantor

Surahman Hakim MD.

Yulia Margaretta Sari MD.

Achmad Kemal Harzif MD.

## Provenance and peer review

Not commissioned, externally peer-reviewed.

## Declaration of Competing Interest

This case report do not have any relationship with other people or organisations.
